# *CYP2E1*, *GSTM1*, and *GSTT1* genetic polymorphisms and their associations with susceptibility to antituberculosis drug-induced liver injury in Thai tuberculosis patients

**DOI:** 10.1016/j.heliyon.2021.e06852

**Published:** 2021-04-20

**Authors:** Noppadol Chanhom, Sukanya Wattanapokayakit, Nusara Satproedprai, Supharat Suvichapanich, Surakameth Mahasirimongkol, Usa Chaikledkaew, Wanvisa Udomsinprasert, Taisei Mushiroda, Jiraphun Jittikoon

**Affiliations:** a Department of Biochemistry, Faculty of Pharmacy, Mahidol University, Bangkok 10400, Thailand; b Department of Pharmacy, Faculty of Pharmacy, Mahidol University, Bangkok 10400, Thailand; c Genomic Medicine Centre, Division of Genomic Medicine and Innovation Support, Department of Medical Sciences, Ministry of Public Health, Nonthaburi 11000, Thailand; d Laboratory for Pharmacogenomics, RIKEN Center for Integrative Medical Sciences, 1-7-22 Suehirocho, Tsurumi-ku, Yokohama, Japan

**Keywords:** Adverse drug reaction, Drug-induced liver injury, Genetic polymorphisms, Glutathione s-transferase, Hepatotoxicity, Tuberculosis

## Abstract

Antituberculosis drug-induced liver injury (ATDILI) is the common adverse reaction of antituberculosis drugs. Glutathione S-transferases (GSTs), which are phase II metabolizing enzymes for detoxification, are recognized as potential mediators of hepatotoxicity. However, role of *GST*s polymorphisms in ATDILI pathogenesis has never been observed in Thais. This study aimed to investigate associations between *GST*s and ATDILI susceptibility. This retrospective case-control multicentered study was conducted by the collaboration from ten secondary and tertiary care hospitals across Thailand, including Northern, Central, and Southern parts of Thailand. We enrolled 80 tuberculosis (TB) patients with ATDILI and 174 those without ATDILI into the study. Polymerase chain reaction (PCR) was used to determine genetic polymorphisms of *GSTM1* and *GSTT1* genes. *CYP2E1* genotyping data were derived from microarray data. We illustrated that *GSTT1* null and *GSTM1*/*GSTT1* dual null genotypes were correlated with an increased risk of ATDILI with odds ratio (OR) at 1.83 (95% confidence interval (CI), 1.00 to 3.35; P = 0.049) and 2.12 (95%CI, 1.02 to 4.38; P = 0.044), respectively. Interestingly, *GSTT1* null and *GSTM1*/*GSTT1* dual null genotypes were found to be correlated with an increased risk of ATDILI in Thai TB patients who carried *CYP2E1* wild type phenotype with OR 2.99 (95%CI, 1.07 to 8.39; P = 0.037) and 3.44 (95%CI, 1.01 to 11.71; P = 0.048), respectively. Collectively, *GSTT1* null and *GSTM1*/*GSTT1* dual null genotypes were associated with a higher risk of ATDILI in Thai TB patients, which may serve as alternative genetic biomarkers for ATDILI.

## 1 Introduction

Hepatotoxicity is the most frequent cause of tuberculosis (TB) treatment discontinuation that increases the incident rate of multi-drug-resistant TB and ultimately leads to treatment failure [1]. Besides treatment discontinuation, the majority of TB patients with drug-induced liver injury (DILI) develop irreversible liver failure and eventually require liver transplant, due to a poorly defined pathogenesis and delayed diagnosis [2]. Unfortunately, ATDILI is difficult to predict and manage, because this adverse drug reaction is considered a multifactorial condition caused by a wide range of risk factors including the dose duration, hepatic metabolism and lipophilicity of the antituberculosis (anti-TB) drugs, and other factors consisting of sex, age, and metabolism of the patients [2]. In particular, genetics has been recognized as a critical contributor to the pathogenesis of DILI [3].

Of various genes known to be associated with ATDILI, *NAT2* has been extensively studied in association with ATDILI. Notably, some specific variants of *NAT2* have been shown to be strongly associated with ATDILI induced by isoniazid (INH) [4, 5]. *NAT2* variants can affect the enzymatic activity and acetylation rate of NAT2. *NAT2* phenotypes can be categorized into four groups including rapid, intermediate, slow, and ultra-slow acetylators [6]. Slow acetylation of INH can result in the increased concentrations of hydrazine and acetylhydrazine, INH toxic metabolites, which increased the toxicity of the drug and subsequently induced the progressive and developmental ATDILI in TB patients [2]. However, some TB patients who carried *NAT2* rapid and intermediate acetylation phenotypes had a possibility of developing ATDILI. As a result, other genes must be taken into account in order to increase ATDILI predictability using a genetic biomarker.

**Figure 1:**
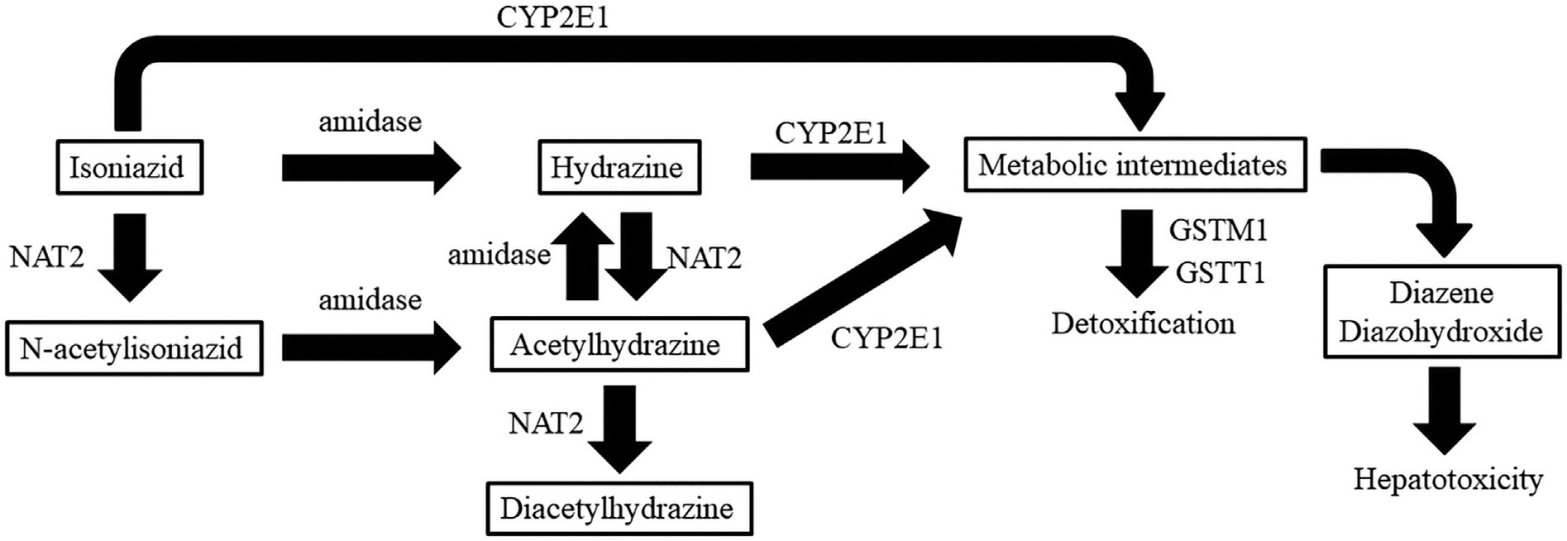
Isoniazid metabolism pathways representing isoniazid metabolites and involved enzymes. This figure was modified from PharmGKB online database [33,34] at http://www.pharmgkb.org/pathway/PA166151813.

GSTs, essential phase II metabolizing enzymes for detoxification, are gaining increasing interest as potential mediators of hepatotoxicity. GSTs are responsible for mitigating the cellular damage resulting from oxidative stress via conjugating glutathione to substrates including reactive oxygen species (ROS), which are metabolites of isoniazid made by CYP2E1 metabolism activity in response to liver damage (Figure 1) [7]. Therefore, GSTs can exert a protective effect against cellular damage. In addition, there is a prior study illustrating that gene deletions caused by the homozygous null mutation of *GSTM1* and *GSTT1*, which are two essential *GST*s involved in the isoniazid metabolism pathway, were significantly associated with a higher risk of ATDILI in patients with TB [8]. Moreover, a number of clinical studies have provided supporting evidences of significant association between *GSTM1* or *GSTT1* and susceptibility to ATDILI [9, 10, 11, 12].

CYP2E1, an essential phase I metabolizing enzyme in isoniazid metabolism pathways, is expressed by the polymorphic *CYP2E1* gene. CYP2E1 is responsible for the metabolism of isoniazid, hydrazine, and acetylhydrazine, which are parent drugs and its two major isoniazid hepatotoxic metabolites (Figure 1). However, according to Wang *et al.*, the product of the CYP2E1 enzyme is also a reactive metabolite [13]. As to a primary role of CYP2E1, several studies have reported that *CYP2E1∗5*, which is one of the *CYP2E1* variant alleles caused by single nucleotide polymorphisms (SNP) at rs2031920 of *CYP2E1* gene [14], was involved in a lower risk of ATDILI in TB patients [8, 15, 16], due to its lower metabolizing activity than wild type allele or *∗1A* [17]. In addition, a study by Wang *et al.* has demonstrated that *CYP2E1*∗*7* (rs2070673) had a higher frequency in the Asian population than that in European, American, or African-American [18]. Based on this previous finding, three alleles of the *CYP2E1* gene, including *∗1A*, *∗5*, and *∗7*, were characterized in this study in order to investigate their associations with ATDILI risk in Thai TB patients.

Although previous studies have reported that *NAT2* slow acetylator status was associated with ATDILI in Thai TB patients [4, 19], to the best of our knowledge, no published studies examined the associations between *GST*s genotype and the risk of ATDILI in Thai population. Furthermore, there are no studies reporting on the combined effect of *GST*s with *CYP2E1* genotypes in predicting the risk ATDILI in Thai population. Accordingly, our study was designed to investigate the associations of *GST*s genotypes and the combined effect of with *GST*s genotypes with *CYP2E1* with ATDILI susceptibility in Thai TB patients.

## 2 Materials and methods

### 2.1 Study subjects

The subjects in this study were Thai TB patients receiving World Health Organization anti-TB regimens category I (2HRZE/4HR) [20] from Bangplama Hospital (Suphan Buri), The Central Chest Disease Institute (Nonthaburi), Chiang Rai Prachanukroh Hospital (Chiang Rai), Hatyai Hospital (Songkla), Maesot Hospital (Tak), Nopparat Rajathanee Hospital (Bangkok), Buddhachinaraj Hospital (Phitsanulok), Ramathibodi Hospital (Bangkok), Rayong Hospital (Rayong), and Thai Mueang Chaipat Hospital (Phang-nga) from the year 2012–2018. This study protocol was performed in compliance with the International Guidelines for Human Research Protection, including the Declaration of Helsinki, the Belmont Report, etc. and was approved by the Institutional Review Board of the Faculty of Dentistry/Faculty of Pharmacy, Mahidol University (IRB No. 2019/024.0205). Written informed consent was obtained from all patients before their admissions to the study.

**Figure 2:**
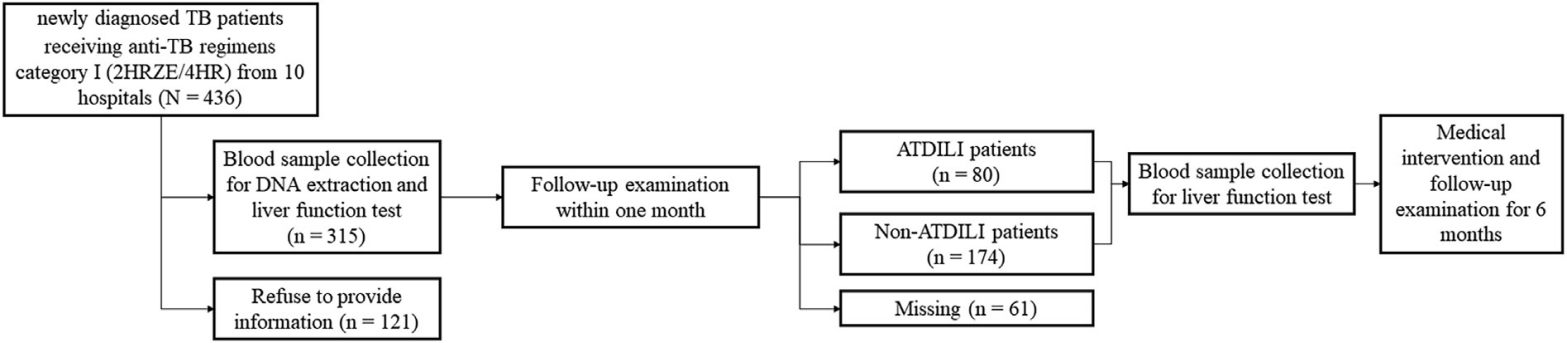
Patient selection process. ATDILI, antituberculosis drug-induced liver injury; E, ethambutol; H, isoniazid; R, rifampicin; TB, tuberculosis; Z, pyrazinamide.

A total of 254 patients diagnosed with TB were recruited into this case-control study (Figure 2). The subjects were categorized into 80 patients with ATDILI and 174 those with non-ATDILI patients according to their clinical symptoms and levels of liver function markers including aspartate aminotransferase (AST), alanine aminotransferase (ALT), and total bilirubin (Tbil). In accordance with clinical practice guideline for TB treatment in Thailand [21], ATDILI patients were defined using one of the following features. (i) AST or ALT levels were higher than three times upper limit of normal (ULN) along with one symptom of hepatitis including anorexia, fatigue, jaundice, liver enlargement, nausea vomiting, or dark urine. (ii) AST or ALT levels were higher than five times ULN or Tbil level higher than three times ULN with or without symptoms of hepatitis. The control or non-ATDILI group was defined by the absence of signs and symptoms of hepatotoxicity during the treatment period. Instead, the patients who (i) have concomitant administration of other potentially hepatotoxic drugs according to LiverTox database [22], (ii) have underlying disease consisting of viral hepatitis, liver cirrhosis, hepatoma, or human immunodeficiency virus infection, and (iii) have cognitive dysfunction were excluded from this study. The clinical information and blood samples of the patients were collected by onsite associates and recorded in specific pre-defined clinical record form of the project.

### 2.2 Assessment of clinical outcomes

Blood samples from all patients were collected in ethylenediaminetetraacetic acid tubes. The collected blood samples were then centrifuged to separate plasma and buffy coat containing a massive amount of leukocytes. The extracted buffy coat and plasma were subsequently stored in -80 °C until analysis. All liver function biomarkers including ALT, AST, ALP, albumin (ALB), Tbil, and direct bilirubin (DB) were measured routinely by an automated machine of each hospital. Levels of AST and ALT were then divided by each hospital's validated-ULN in order to monitor hepatotoxicity status according to the mentioned criteria.

### 2.3 DNA extraction

Genomic DNA was extracted from peripheral blood leukocytes in the patient's venous blood using a QIAamp® DNA Mini Kit (QIAGEN, CA, USA) or from the patient's buccal cells from saliva sample using Geneaid DNA Mini Kit (Geneaid Biotech, Taiwan), according to manufacturer's protocol. Extracted DNA was further analyzed using agarose gel electrophoresis and quantitatively determined by ultraviolet spectrometer NanoDrop 2000c (Thermo Scientific, MA, USA).

### 2.4 Genotyping

*GSTM1* and *GSTT1* genotypes were determined using PCR. β-globin was used as an internal standard. The primers used for *GSTM1* and *GSTT1* were, as follows: *GSTM1* forward 5′–GAACTCCCTGAAAAGCTAAAGC–3'; *GSTM1* reverse 5′– GTTGGGCTCAAATATACGGTGG–3'; *GSTT1* forward 5′– TTCCTTACTGGTCCTCACATCTC–3'; *GSTT1* reverse 5' – TCACCGGATCATGGCCAGCA– 3' [23]. The PCR was performed with 20 ng of genomic DNA in a total volume of 20 μl using T100 Thermal Cycler (BIORAD, CA USA) and KAPA2G Fast Multiplex PCR Kit (KAPA Biosystems, MA, USA) with initial denaturation at 95 °C for 5 min, followed by 35 cycles of denaturation at 95 °C for 15 s, annealing at 63 °C for 20 s, and extension at 72 °C for 20 s. The PCR products were analyzed on 2% agarose gel pre-stained with ethidium bromide. The *GSTM1* gene was identified by the presence or absence of bands at 219 base pairs, whereas the *GSTT1* gene was interpreted by the presence or absence of bands at 459 base pairs. *CYP2E1* genotyping data were received and extracted from previous results derived from microarray in a previously published article by Suvichapanich *et al.* [19] and partially additional data from Illumina Infinium Asian Screening Array-24 version 1.0 BeadChip (Illumina, San Diego, CA) microarray (Table S1).

### 2.5 Data analysis

Genotyping data at SNPs rs2031920 and rs2070673 were extracted from microarray data in order to identify *CYP2E1*∗*5* and ∗*7*, respectively. Haplotype reconstruction was performed by PHASE v2.1.1 software [24, 25]. *CYP2E1* genotypes were inferred from reconstructed haplotype in consonance with The Pharmacogene Variation Consortium [26]. Hardy-Weinberg equilibrium was tested using χ^2^. The statistical analyses were conducted by the statistical package for social sciences version 22.0 (SPSS, Inc., IL, USA). Quantitative parameters such as demographics and clinical data were compared between groups using χ^2^ tests and Student's *t*-test, where appropriate. Comparisons among each group were evaluated by the Mann-Whitney *U* test or Kruskal-Wallis *H* test. The genotypic distribution was compared between cases and controls using Fisher's exact test. To control the role of confounding factors including age and gender, binary logistic regression was used. For all analyses, a two-tailed P-value of less than 0.05 was considered to be statistically significant.

## 3 Results

### 3.1 Demographic and clinical characteristics of TB patients

As described in Table 1, there were significantly differences in baseline demographic data and clinical characteristics between TB patients with and without ATDILI. Mean age of TB patients with ATDILI (52.6 ± 17.3 years) was significantly higher than that of TB patients without ATDILI (46.2 ± 14.8 years) (P = 0.007). Furthermore, gender ratio were significantly higher than in the patients with ATDILI (male: female; 51.25%: 48.75%) than that in the non-ATDILI patients (male: female; 66.67%: 33.33%) (P = 0.026). Additionally, clinical parameters including AST, ALB, and Tbil measured within seven days after treatment initiation (baseline) were significantly greater in TB patients with ATDILI than that in those with non-ATDILI (P < 0.021, P=<0.001, P < 0.004, respectively). As expected, all of liver function markers including ALP, AST, ALT, ALB, Tbil, and DB assessed within sixty days after treatment initiation were considerably increased in ATDILI patients, compared with those without ATDILI (P < 0.001).

**Table 1:** Clinical characteristics of tuberculosis patients with and without ATDILI within seven days and 60 days after anti-TB drugs treatment initiation.

Variables	Within 7 days after treatment		P-value	Within 60 days after treatment		P-value
	ATDILI (n = 80)	Non-ATDILI (n = 174)		ATDILI (n = 80)	Non-ATDILI (n = 174)	
ALP (IU/L)	144 ± 122	98.3 ± 49.4	0.066	193 ± 185	84.5 ± 41.2	**<0.001**
AST (IU/L)	54.7 ± 71.1	31.3 ± 22.8	**0.021**	249 ± 269	31.2 ± 22.3	**<0.001**
ALT (IU/L)	48.2 ± 63.5	23.7 ± 27.5	0.143	156 ± 135	28.5 ± 28.0	**<0.001**
ALB (g/dL)	3.33 ± 0.78	3.86 ± 0.41	**<0.001**	3.29 ± 0.74	4.02 ± 0.41	**<0.001**
TBil (mg/dL)	1.77 ± 2.89	0.59 ± 0.36	**0.004**	2.88 ± 4.02	0.52 ± 0.23	**<0.001**
DB (mg/dL)	0.83 ± 1.72	0.20 ± 0.15	0.064	1.85 ± 2.96	0.18 ± 0.13	**<0.001**

Abbreviation: ALB, albumin; ALP, alkaline phosphatase; ALT, alanine aminotransferase; AST, aspartate aminotransferase; ATDILI, anti-tuberculosis drug-induced liver injury; DB, direct bilirubin; TBil, total bilirubin. Significant results with P-value less than 0.05 were highlighted in bold.

### 3.2 Associations of *GSTM1* and *GSTT1* genotypes with susceptibility to ATDILI

Genotypic distribution of GSTs in non-ATDILI and ATDILI patients is shown in Table 2. *GSTM1* homozygous null genotype was found to be more prevalent in TB patients with ATDILI; however, there was no association between *GSTM1* homozygous null genotype and ATDILI risk in TB patients. On the contrary, *GSTT1* homozygous null genotype and *GSTM1*/*GSTT1* dual null genotypes were significantly associated with ATDILI risk in TB patients, after adjusting for age and sex (adjusted OR = 1.83; 95%CI, 1.01 to 3.35, P = 0.049; adjusted OR = 2.12; 95%CI, 1.02 to 4.38, P = 0.044, respectively).

**Table 2:** Association between *GSTM1* and *GSTT1* genotype and susceptibility to ATDILI.

*GSTs*	ATDILI (n = 80)	Non-ATDILI (n = 174)	OR (95% CI)	P-value	Adjusted OR (95% CI)	P-value
*GSTM1* null	47 (58.8%)	97 (55.7%)	1.131 (0.661–1.933)	0.684	1.103 (0.633–1.921)	0.73
*GSTT1* null	28 (35.4%)	40 (23.0%)	1.839 (1.029–3.287)	0.047∗	1.834 (1.004–3.352)	0.049∗
*GSTM1/GSTT1* dual null	18 (22.8%)	20 (11.5%)	2.272 (1.126–4.587)	0.024∗	2.115 (1.021–4.383)	0.044∗

Adjusted for age and gender.

Abbreviation: ATDILI, anti-tuberculosis drug-induced liver injury; CI, confidence interval; GSTM1, glutathione S-transferase mu 1; GSTs, glutathione S-transferase mu 1 and glutathione; S-transferase theta 1; GSTT1, glutathione S-transferase theta 1; OR, odd ratio.

### 3.3 Association between *CYP2E1* polymorphisms and ATDILI susceptibility

In addition to *GSTM1* and *GSTT1* polymorphisms, the distribution of *CYP2E1* genetic polymorphisms in TB patients with and without ATDILI was further determined, in which *CYP2E1* genotypes were derived from both a previous study by Suvichapanich *et al.* [19] and additional data from Illumina Infinium Asian Screening Array-24. The distribution of SNP genotypes among 254 TB patients was consistent with Hardy-Weinberg equilibrium (P > 0.05). The allelic distribution of two SNPs within *CYP2E1* gene is summarized in Table 3. None of the SNPs was observed to be associated with ATDILI susceptibility. Consistent with SNPs of *CYP2E1*, there were no associations between *CYP2E1* haplotypes and risk of ATDILI (Table 4).

**Table 3:** *CYP2E1* SNP-based allelic association tests in Thai TB patients with and without ATDILI.

SNP ID	Allele 1/2	Risk allele^a^	ATDILI (n = 80)				Non-ATDILI (n = 174)				OR (95%CI)^b^	P-value	HWE		
			11 n (%)	12 n (%)	22 n (%)	RAF %	11 n (%)	12 n (%)	22 n (%)	RAF %			Case	Control	All
rs2031920	C/T	C	54 (67.5)	24 (30)	2 (2.5)	0.825	120 (69)	47 (27)	7 (4)	0.825	1.000 (0.612–1.640)	0.994	0.121	0.752	0.273
rs2070673	T/A	A	23 (28.8)	42 (52.5)	15 (18.8)	0.45	59 (33.9)	87 (50)	28 (16.1)	0.41	1.173 (0.804–1.711)	0.409	0.294	0.187	0.412

Abbreviation: ATDILI, anti-tuberculosis drug-induced liver injury; CI, confidence interval; HWE, Hardy-Weinberg equilibrium; OR, odd ratio; RAF, risk allele frequency; SNP, single nucleotide polymorphisms.

a Defined as alleles with higher frequency in individuals with DILI than tolerant controls.

b Calculated for allelic model.

**Table 4:** Distribution of *CYP2E1* haplotypes in Thai TB patients with and without ATDILI.

Haplotype		ATDILI (2n = 160)	Non-ATDILI (2n = 348)	OR (95%CI)^a^	P-value
*∗1A*	C-T	88 (55.0%)	205 (58.9%)	0.853 (0.584–1.244)	0.408
*∗5*	T-A	28 (17.5%)	61 (17.5%)	0.998 (0.610–1.633)	0.994
*∗7*	C-A	44 (27.5%)	82 (23.6%)	1.230 (0.803–1.885)	0.340

Abbreviation: ATDILI, anti-tuberculosis drug-induced liver injury; CI, confidence interval; OR, odd ratio.

a Calculated for haplotype frequencies.

### 3.4 Combination of phenotypic distribution of *CYP2E1* and genotypic distribution of *GST*s in non-ATDILI and ATDILI patients

We additionally performed a subgroup analysis according to *CYP2E1* phenotypes and *GST*s genotypes of the patients. As demonstrated in Table 5, *GSTT1* null genotype was found to be significantly associated with a 2.99-fold increased risk of ATDILI in the TB patients who carried *CYP2E1* wild type (adjusted OR = 2.99; 95% CI, 1.07 to 8.40; P = 0.037). Consistent with *GSTT1* polymorphisms, we also observed the significant association between *GSTM1*/*GSTT1* dual null polymorphisms and an increased risk of ATDILI in TB patients who carried *CYP2E1* wild type (adjusted OR = 3.44; 95% CI, 1.01 to 11.71; P = 0.048). For the *GSTM1* null genotype, there was no correlation with ATDILI susceptibility in the patients who carried all of *CYP2E1* phenotypes.

**Table 5:** Association between combination of *CYP2E1* and *GSTT1* and susceptibility to ATDILI.

Phenotypes	GST genotypes	ATDILI	Non-ATDILI	OR (95%CI)	P-value	Adjusted OR (95%CI)	P-value
CYP2E1 Wild type	GSTM1 null	15	33	1.48 (0.54–4.02)	0.468	1.53 (0.55–4.24)	0.417
	GSTT1 null	12	17	2.97 (1.08–8.15)	0.04	2.99 (1.07–8.39)	0.037
	GSTM1/GSTT1 dual null	7	7	3.47 (1.05–11.45)	0.049	3.44 (1.01–11.71)	0.048
CYP2E1 Variant	GSTM1 null	32	64	1.02 (0.54–1.93)	1	1.05 (0.53–2.08)	0.894
	GSTT1 null	16	23	1.56 (0.75–3.26)	0.25	1.68 (0.76–3.70)	0.198
	GSTM1/GSTT1 dual null	11	13	1.88 (0.78–4.50)	0.167	1.85 (0.73–4.73)	0.198

Adjusted for age and gender.

CYP2E1 wild type = *∗1A*/*∗1A*.

CYP2E1 variant = *∗1A*/*∗5*, *∗1A*/*∗7*, *∗5*/*∗5*, *∗5A*/*∗7*, *∗7*/*∗7*.

Abbreviation: ATDILI, anti-tuberculosis drug-induced liver injury; CI, confidence interval; OR, odd ratio.

## 4 Discussion

Since the discovery, anti-TB drugs have been holding the key thwart spread of TB disease. However, the ADRs caused by these anti-TB drugs have become a new nemesis of TB eradication. Among all types of ADRs caused by anti-TB medication, hepatotoxicity, also known as ATDILI, has a high prevalence [27] and mortality [28]. In addition, these worrying ADRs escalate a multi-drug resistance TB incident rate and occasionally lead to TB treatment failure [29]. Therefore, in order to terminate TB infection, ATDILI prevention is an unavoidable concern. Doubtlessly, reliable and specific biomarkers for ATDILI predictions are in urgent demand. Genetic variations associated with ATDILI have been extensively studied in numerous populations. They may potentially serve as genetic biomarkers for identifying patients with a high risk of developing ATDILI prior to the prescription of anti-TB drugs or regimens, which may exacerbate the ATDILI progression [30]. In support of this hypothesis, two studies [4,19] have unveiled the association between *NAT2* genetic polymorphisms and the risk of ATDILI in Thai TB patients. Based on this premise, *NAT2* genetic polymorphisms can be considered as potential genetic biomarkers for predicting ATDILI progression. However, *NAT2* polymorphisms were able to identify about 70 percent of ATDILI patients, but not all of TB patients with ATDILI [4]. From this, another genetic biomarker is required to improve the ATDILI predictability. GST enzymes, which are essential detoxification enzymes against the production of ROS and reactive metabolites, may be potential candidates as genetic biomarkers for predicting the risk of ATDILI in Thai TB patients.

In the present study, we found that ATDILI patients had significantly increased values of AST, ALB, and Tbil measured within seven days after treatment initiation than those without ATDILI. In addition, the results of reassessed liver enzymes within 60 days after treatment initiation illustrated that the ATDILI patients had significantly higher levels of liver function markers including ALP, AST, ALP, ALB, Tbil, and DB than the non-ATDILI patients.

In terms of genetic polymorphisms, we determined the associations between *GST*s polymorphisms and the risk of ATDILI. We also found that *GSTT1* homozygous null genotype and *GSTM1*/*GSTT1* dual null genotype were correlated with the risk of ATDILI in Thai TB patients. As mentioned above, *GSTM1* and *GSTT1* are two essential *GST*s involved in the isoniazid metabolism pathway. Homozygous deletion of *GSTM1* and *GSTT1* gene can cause the absence of GSTM1 and GSTT1 enzymes, respectively, and subsequent absence of glutathione conjugation activity. As a result of the lack of glutathione conjugation activity, the liver cells are prone to be damaged by oxidative stress and isoniazid reactive metabolites [31]. In this study, we found that *GSTT1* null and *GSTM1/GSTT1* dual null genotypes were both correlated with an increased risk of ATDILI in Thai TB patients. These results were in line with several studies demonstrating the associations between *GSTs* null genotypes and an increased risk of ATDILI [8, 9, 11].

Attesting the relationships between GSTs and other drug-metabolizing enzymes, a previous study by Chanhom *et al.* involving protein-protein interaction analysis has uncovered that there were direct links between *NAT2*, *CYP2E1,* and *GST*s. From this finding, it has been hypothesized that genetic polymorphisms within those genes may be implicated in ATDILI [32]. To address this speculation, the combination analysis of *CYP2E1* phenotypic polymorphisms and *GSTs* genetic polymorphisms was investigated. In our subgroup analyses based on *CYP2E1* polymorphisms, we found that *GSTT1* homozygous null genotype and *GSTM1*/*GSTT1* dual null genotype were both associated with the risk of ATDILI in *CYP2E1* wild type allele group. These findings support the notion that *GSTM1* and *GSTT1* genetic polymorphisms may have the potential as a genetic biomarker for ATDILI progression in TB patients – especially the patients who carried *CYP2E1* wild type. According to isoniazid metabolism pathways [33, 34], GST enzymes are supposed to detoxify the reactive intermediate metabolites produced by CYP2E1 enzyme oxidation activity of isoniazid, hydrazine, and acetylhydrazine [35] being the parent drug and two of its metabolites produced by NAT2 and amidase enzymes (Figure 1). In regard to their general role, GST enzymes are used to metabolize the reactive metabolites produced by CYP2E1, NAT2, and other enzymes. This might be the reason why *CYP2E1* alone was not associated with the risk of ATDILI in this study and several reports [36, 37, 38], because the toxic metabolites could be produced by several molecules mediated through several pathways. Attesting this speculation, in the current study, the combination analysis of *CYP2E1* phenotypic polymorphisms and *GSTs* genetic polymorphisms provided clear results of their associations with ATDILI. In subgroup analysis of *CYP2E1* genotypes in TB patients, we found that *GSTT1* null genotype and *GSTM1*/*GSTT1* dual null genotype were both associated with ATDILI in TB patients who carried *CYP2E1* wild type allele. All of these results would be supporting evidence that the risk of ATDILI is influenced by multi-genetic contributions. Therefore, further study should concern the multi-genetic effects on the risk of ATDILI including *NAT2*, *CYP2E1*, and *GST*s genetic polymorphisms.

There are certain limitations to this study. The most important drawback of the present study is its study design, in which this is a retrospective case-control study preventing the determination of cause-and-effect relationships. It is recommended that multi-center prospective cohorts are needed to verify any associations. Furthermore, this study was unable to identify the individual drugs-induced liver injury, given that TB treatment guidelines [21] indicate all four anti-TB drugs simultaneously administered to TB patients, and rechallenge histories were missing in many cases. Since we extract the data from microarray results, we cannot determine SNP rs6413420 located in the intron region of the *CYP2E1* gene. Therefore, we cannot identify the *CYP2E1*∗*6* allele and separate *CYP2E1*∗*5* to *CYP2E1*∗*5A* and *CYP2E1*∗*5B*. Third, we did not include the effect of *NAT2* genetic polymorphisms combined with *CYP2E1* and *GST*s polymorphisms on ATDILI in our study. Another limitation is the fact that we were unable to determine whether the initial treatment affected the significant results presented in our study, due to unavailability of the information. Although the initial treatment and retreatment may have an impact on ATDILI development in TB patients, it has been well-recognized that genetic polymorphisms were not influenced by extrinsic factors. For this reason, our significant results regarding association between *GSTs* polymorphisms and ATDILI risk in TB patients remain unchanged. Finally, as we concerned, Roussel Uclaf Causality Assessment Method (RUCAM) is exceptional for the DILI causality assessment method [39], and we have already considered the method to be applied in our study. However, according to the national guideline for tuberculosis treatment in Thailand, which agrees with WHO guidelines for tuberculosis treatment, the Thai guideline indicates that if the patient has the symptom of hepatitis with AST/ALT ≥3 ULN, the physician should stop the medication and intervention must take place. Our study's strength is that we can use the information to prevent and reduce the possibility of ATDILI cases in Thai TB patients. Additionally, we investigated the multi-genetic effects on ATDILI, which may facilitate the development of personalized treatment strategies in the upcoming future.

## 5 Conclusion

Collectively, our study revealed that both *GSTT1* null genotype and *GSTM1*/*GSTT1* dual null genotype were associated with the risk of ATDILI in Thai TB patients. Furthermore, our study illustrated that the combination of *CYP2E1* and *GST*s genotypes was associated with susceptibility to ATDILI, which might be useful for predicting the risk of ATDILI in TB patients. In order to draw a more precise conclusion and support the use of *GSTT1* as an additional genetic biomarker for predicting the risk of ATDILI in TB patients, further studies are warranted to investigate multi-genetic effects including *NAT2*, *CYP2E1*, and *GSTs* genetic polymorphisms on the risk of ATDILI.

## Declarations

### Author contribution statement

Noppadol Chanhom: Conceived and designed the experiments; Performed the experiments; Analyzed and interpreted the data; Wrote the paper.

Sukanya Wattanapokayakit: Conceived and designed the experiments; Performed the experiments; Contributed reagents, materials, analysis tools or data.

Nusara Satproedprai: Performed the experiments; Contributed reagents, materials, analysis tools or data.

Supharat Suvichapanich: Analyzed and interpreted the data.

Surakameth Mahasirimongkol: Conceived and designed the experiments; Contributed reagents, materials, analysis tools or data.

Usa Chaikledkaew: Conceived and designed the experiments.

Wanvisa Udomsinprasert, Jiraphun Jittikoon: Conceived and designed the experiments; Analyzed and interpreted the data; Wrote the paper.

Taisei Mushiroda: Contributed reagents, materials, analysis tools or data.

### Funding statement

This work was supported by the International Research Network - The Thailand Research Fund (IRN60W003); e-ASIA Joint Research Program (the e-ASIA JRP); 10.13039/501100010724Health Systems Research Institute; and Medical Scholar Program of Mahidol University.

### Data availability statement

Data included in article/supplementary material/referenced in article.

### Declaration of interests statement

The authors declare no conflict of interest.

### Additional information

No additional information is available for this paper.

## Appendix A Supplementary data

Manuscript_RV3_V1.docx(Page no 29) Manuscript_RV3_V1.docx(Page no 29)
