# Supplementary material for: CYP2E1, GSTM1, and GSTT1 genetic polymorphisms and their associations with susceptibility to antituberculosis drug-induced liver injury in Thai tuberculosis patients
Source: Heliyon. 2021 Apr 20;7(4):e06852. doi: 10.1016/j.heliyon.2021.e06852 (PMC8082558; doi:10.1016/j.heliyon.2021.e06852)
Supplement: Manuscript_RV3_V1.docx(Page no 29) [file mmc1.docx]

**Supplementary**

**Table S1**. *CYP2E1* data acquiring and haplotype frequencies

|  | Received from | | Extracted from microarray data^a^ | |
| --- | --- | --- | --- | --- |
|  | Suvichapanich *et al*. (20) | |  |  |
|  | ATDILI | Non-ATDILI | ATDILI | Non-ATDILI |
| Number of Samples | 46 | 174 | 34 | 0 |
| Haplotype frequencies |  |  |  |  |
| **1A* | 52 | 205 | 36 | 0 |
| **5* | 15 | 61 | 13 | 0 |
| **7* | 25 | 82 | 19 | 0 |

^a^ Data from Illumina Infinium Asian Screening Array-24 version 1.0 BeadChip Microarray
